# Molecular Epidemiology of *mcr*-Encoded Colistin Resistance in *Enterobacteriaceae* From Food-Producing Animals in Italy Revealed Through the EU Harmonized Antimicrobial Resistance Monitoring

**DOI:** 10.3389/fmicb.2018.01217

**Published:** 2018-06-12

**Authors:** Patricia Alba, Pimlapas Leekitcharoenphon, Alessia Franco, Fabiola Feltrin, Angela Ianzano, Andrea Caprioli, Fiorentino Stravino, Rene S. Hendriksen, Valeria Bortolaia, Antonio Battisti

**Affiliations:** ^1^Department of General Diagnostics, National Reference Laboratory for Antimicrobial Resistance, Istituto Zooprofilattico Sperimentale del Lazio e della Toscana, Rome, Italy; ^2^WHO Collaborating Centre for Antimicrobial Resistance in Foodborne Pathogens and Genomics and European Union Reference Laboratory for Antimicrobial Resistance, National Food Institute, Technical University of Denmark, Kongens Lyngby, Denmark

**Keywords:** epidemiology, colistin resistance, *mcr* genes, whole genome sequencing, food-producing animals, Italy, *E. coli*, *Salmonella*

## Abstract

Colistin resistance by mobilisable *mcr* genes has been described in bacteria of food-animal origin worldwide, which has raised public health concerns about its potential foodborne transmission to human pathogenic bacteria. Here we provide baseline information on the molecular epidemiology of colistin-resistant, *mcr*-positive *Escherichia coli* and *Salmonella* isolates in food-producing animals in Italy in 2014-2015. A total 678, 861 and 236 indicator *E. coli*, Extended Spectrum Beta-Lactamase (ESBL)/AmpC-producing *E. coli*, and *Salmonella* isolates, respectively, were tested for colistin susceptibility. These isolates were collected according to the EU harmonized antimicrobial resistance monitoring program and are representative of at least 90 and 80% of the Italian poultry (broiler chickens and turkeys) and livestock (pigs and bovines < 12 months) production, respectively. Whole genome sequencing by Illumina technology and bioinformatics (Center for Genomic Epidemiology pipeline) were used to type 42 *mcr-*positive isolates by PCR. Colistin resistance was mainly observed in the ESBL/AmpC *E. coli* population, and was present in 25.9, 5.3, 6.5, and 3.9% of such isolates in turkeys, broilers, pigs, and bovines, respectively. Most colistin-resistant isolates (141/161, 87.5%) harbored genes of the *mcr*-1 group. *mcr-*1 was also detected in a small proportion of *Salmonella* isolates (3/146, 2.0%) in turkeys. Additional *mcr* types were *mcr*-3 in four ESBL-producing *E. coli* from bovines, and two *mcr*-4 in ESBL (*n* = 1) and indicator *E. coli* (*n* = 1) from pigs and bovines. We describe notable diversity of *mcr* variants with predominance of *mcr-*1.1 and *mcr-*1.2 on conjugative IncX4 plasmids in *E. coli* and in *Salmonella* serovars Typhimurium, Newport, Blockley from turkey. A new variant, *mcr*-1.13 was detected in the chromosome in *E. coli* in turkey and pig isolates. Additionally, we describe *mcr*-3.2 and *mcr*-4.3 in *E. coli* from bovines, and *mcr*-4.2 in *E. coli* from pigs. These findings elucidate the epidemiology of colistin resistance in food-producing animals in Italy along with its genetic background, and highlight the likelihood of *mcr* horizontal transfer between commensal bacteria and major food-borne pathogens (*Salmonella*) within the same type of productions. Thorough action and strategies are needed in order to mitigate the risk of *mcr* transfer to humans, in a “One Health” perspective.

## Introduction

Colistin is a polymyxin classified among the Highest Priority Critically Important Antimicrobials for human medicine by the World Health Organization (WHO), and it is considered a last resort antimicrobial for the treatment of infections by carbapenem-resistant *Enterobacteriaceae* in humans (Poirel et al., 2017).

In 2015, colistin resistance mediated by *mcr-*1, a phosphoethanolamine transferase gene located on a transferable plasmid was first reported in *Escherichia coli* from animals, food and patients from China (Liu et al., 2016). Since then, at least 32 countries from the five continents have found *mcr-*1 in *E. coli* isolates from different sources including humans, animals and foods (Xavier et al., 2016).

In Europe, the presence of *mcr-*1 was first detected in *E. coli* from poultry meat and humans in Denmark (Hasman et al., 2015). Subsequently, this gene was found in *Enterobacteriaceae* from different sources in almost all European countries, including Italy (Battisti, 2016; Cannatelli et al., 2016). In 2016, a new *mcr* gene, *mcr-*2, was described in *E. coli* in calves and piglets in Belgium (Xavier et al., 2016), followed by the description of three additional mobile colistin resistance genes in 2017, namely *mcr-*3 (Yin et al., 2017) in *E. coli* isolated from pig in China, *mcr-*4 (Carattoli et al., 2017) in *Salmonella* and *E. coli* from pigs in Italy, Spain and Belgium, and *mcr*-5 (Borowiak et al., 2017) in *Salmonella* Paratyphi B from poultry and environmental sources from Germany.

At present, eleven *mcr-*1 variants (KP347127, KX236309, KU934208, KY041856, KY283125, KY352406, KY488488, KY683842, KY964067, KY853650, and LC337668), one *mcr-*2 variant (LT598652), six *mcr-*3 variants (KY924928, NPZH01000177, FLXA01000011, MF463699, MG214533, and MG489958), three *mcr-*4 *variants* (MF543359, MG581979, and ERS1801979) and *mcr*-5 (KY807921) have been described in *Enterobacteriaceae* according to GenBank records (last accessed 21st February 2018).

It is clear that the epidemiology of transferable *mcr*-mediated colistin resistance is evolving rapidly and timely information on prevalence and molecular epidemiology of *mcr-*positive isolates is needed to enhance surveillance, and implement measures to prevention and to control further spread of colistin resistance. In the European Union (EU), the harmonized antimicrobial resistance (AMR) monitoring and reporting in poultry and livestock, which includes colistin susceptibility testing in *E. coli* and *Salmonella*, ensures that prevalence of colistin-resistant bacteria in a representative proportion of the food-animal population is reported from each Member State (MS) (Decision 2013/652/EU). However, the lack of molecular data limits the epidemiologic monitoring of colistin resistance (Schrijver et al., 2017).

The aim of this study is to determine the prevalence of colistin resistance, and the molecular epidemiology of *mcr*-mediated colistin resistance genes and their genetic environment in commensal *E. coli*, Extended Spectrum Beta-Lactamase (ESBL)/AmpC-producing *E. coli*, and *Salmonella* spp. in food-animals in Italy in 2014-2015.

## Materials and Methods

### Study Design, Sample Collection, Isolation, and Identification of Bacterial Cultures

Study design and sampling were performed according to Decision 2013/652/EU^1^, which mandates each EU Member State (MS) to collect caecal content samples from different epidemiological units of poultry flocks (broiler chickens, fattening turkeys), fattening pigs and bovines < 12 months at slaughter.

Samples were collected from broiler chickens (*n* = 300) and fattening turkeys (*n* = 300) in 2014, and from fattening pigs (*n* = 304) and bovines < 12 months (*n* = 223) in 2015 (**Table 1**). The regional stratification of samples represented at least 90 and 80% of the Italian poultry (broiler chickens and turkeys) and livestock (pigs and bovines < 12 months) production, respectively.

**Table 1:** Colistin-resistance and *mcr* genes in *Escherichia coli* and *Salmonella* sp. from caecal samples in animal primary productions, Italy, 2014–2015.

Animal production	Bacterial species	Year	Samples (n)	Isolates tested (n)	Colistin R (n; %)	MIC range mg/L (mode)	*mcr-*1 pos. (n)	*mcr-*2 pos. (n)	*mcr-*3 pos. (n)	*mcr-*4 pos. (n)	*mcr-*5 pos. (n)
Fattening turkeys	Indicator *E. coli*	2014	300	170	39 (22.9%)	4-16 (8)	38	0	0	0	0


	ESBL/*AmpC* *E. coli*	2014	300	224	58 (25.9%)	4-16 (4)	58	0	0	0	0
	*Salmonella* spp.	2014	558	146	12 (8.3%)	4-16 (4)	3	0	0	0	0
Broiler chickens	Indicator *E. coli*	2014	300	170	9 (5.9%)	4-8 (4)	8	0	0	0	0
	ESBL/*AmpC* *E. coli*	2014	300	244	13 (5.3%)	4-16 (4)	11	0	0	0	0
	*Salmonella* spp.	2014	709	90	0	–	–	–	–	0	0
Fattening pigs	Indicator *E. coli*	2015	304	168	1 (0.6%)	4 (4)	1	0	0	0	0
	ESBL/*AmpC* *E. coli*	2015	304	214	14 (6.5%)	4-8 (4)	13	0	0	1	0
Bovine animals <12 months	Indicator *E. coli*	2015	223	170	8 (4.7%)	4-8 (4)	5	0	0	1	0


	ESBL/*AmpC* *E. coli*	2015	223	179	7 (3.9%)	4-8 (4)	4	0	4	0	0


In addition, 558 and 709 samples from fattening turkey and broiler chicken flocks, respectively, were collected within the voluntary national *Salmonella* monitoring framework (Decision 2013/652/EU) in 2014 (**Table 1**). In compliance with Decision 2013/652/EU, *E. coli* isolation and identification, were performed according to the EURL-AR protocols^2^, whereas *Salmonella* spp., isolation, identification and serotyping were performed according to the ISO 6579:2002/Amd 1:2017 protocols.

### Antimicrobial Susceptibility Testing

Antimicrobial susceptibility was tested by minimum inhibitory concentration (MIC) determination using the broth microdilution method, and consensus 96-well microtiter plates (TREK Diagnostic Systems, Westlake, OH, United States). Antimicrobials tested, dilution ranges and interpretation of MIC values were in accordance with Decision 2013/652/EU.

### *mcr* and ESBL/*AmpC* Genes Screening

The presence of *mcr* was investigated in all isolates displaying colistin MIC above the epidemiological cut-off (i.e., > 2 mg/L). A multiple PCR was used to detect *mcr*-1, *mcr*-2, *mcr*-*3*, *mcr*-4, and *mcr*-5 (Rebelo et al., 2018).

ESBL/AmpC-producing *E. coli* positive for *mcr* were further screened for *bla*_CTX-M_, *bla*_SHV_, *bla*_TEM_, *bla*_OXA_, *bla*_CMY_*_-_*_1_, and *bla*_CMY -2_ using primers and PCR conditions previously described (Donati et al., 2014; Franco et al., 2015). Obtained amplicons were Sanger sequenced and analyzed as previously described (Donati et al., 2014; Franco et al., 2015).

### Conjugation Experiments

Four representative *E. coli* and three *Salmonella* sp. isolates from turkeys were selected as donors. Conjugation experiments were performed as previously described (Franco et al., 2015), with the only modification regarding the MacConkey agar plates selective for transconjugants which contained 2 mg/L colistin sulfate and 100 mg/L rifampicin in this study.

### Whole Genome Sequencing (WGS) and Bioinformatics Analysis

A total 42 isolates which tested *mcr*-positive by PCR, (28 *E. coli* and three *Salmonella enterica* isolates from turkeys, five *E. coli* from pigs and six *E. coli* from cattle), and the seven *E. coli* K-12 isolates result of the conjugation experiments were Whole Genome Sequenced. Genomic DNA was extracted using the QIAamp DNA Mini Kit (Qiagen, Hilden, Germany) following the manufacturer’s protocol. Libraries were prepared for Illumina pair-end sequencing using the Illumina (Illumina, Inc., San Diego, CA, United States) NexteraXT^®^ Guide 150319425031942. Sequencing was performed using an Illumina platform (MiSeq or HiSeq2000). Raw sequence data were submitted to the European Nucleotide Archive^3^ under study accession no.: PRJEB23728, PRJEB23778, PRJEB21546, and PRJEB26479.

Raw reads were assembled and analyzed using the pipeline from the Center for Genomic Epidemiology (CGE^4^, Thomsen et al., 2016), with default settings. This pipeline performed *de novo* assembly (Velvet based), species identification (KmerFinder 2.1), Multilocus Sequence Typing (MLST 1.6), identification of virulence (VirulenceFinder 1.2) and antimicrobial resistance genes (ResFinder 2.1), identification of plasmid incompatibility groups (PlasmidFinder 1.2) and plasmid MLST (pMLST 1.4). When identity values for *mcr* were less than 100% in the ResFinder output, the sequence was submitted to online BLAST^5^ (Zhang et al., 2000) to identify the exact *mcr* variant.

Manual annotation of the contigs containing selected *mcr* variants was performed using RAST (Aziz et al., 2008), BLAST (Zhang et al., 2000) and ISfinder (Siguier et al., 2006). The contigs harboring the new *mcr* variants were compared with reference sequences using BLAST and EasyFig (Sullivan et al., 2011). The references sequences used for comparison were CP016034, KP347127 and KY924928.

## Results

### Colistin Resistance and *mcr* in *E. coli* and *Salmonella* From Turkeys and Broilers in 2014

In 2014, colistin resistance was detected in 25.9% (58/224) and 5.3% (13/244) of the ESBL/AmpC-producing *E. coli* from fattening turkeys and broilers, respectively. In turkey flocks, all but two colistin-resistant *E. coli* were multidrug-resistant (MDR) isolates, (i.e., resistant to three antimicrobial classes). In MDR ESBL/AmpC-producing *E. coli* population of turkey flocks, colistin resistance was associated with concurrent fluoroquinolone microbiological resistance (ciprofloxacin MIC >0.064 mg/L) in 51 of 58 isolates (87.9%), with 35/58 (60.3%) displaying fluoroquinolone clinical resistance (MIC >1 mg/L, mode 8 mg/L) (Supplementary Table 1).

A similar prevalence was observed among the indicator commensal *E. coli,* with colistin resistance occurring in 22.9% (39/170) and 5.9% (9/170) of isolates from turkeys and broilers, respectively (**Table 1**). Nearly all colistin-resistant *E. coli* from turkey and broilers tested PCR-positive for *mcr-*1 independent of colistin MIC (**Table 1**).

In 2014, colistin resistance was detected in 8.3% (12/146) and 0% (0/90) of *Salmonella* spp. isolates from fattening turkeys and broiler chickens, respectively. *mcr*-1 was detected only in three of twelve isolates, which displayed colistin MIC = 8 mg/L. The remaining isolates (*n* = 9) did not yield any *mcr* amplicon (**Table 1**).

### Colistin Resistance and *mcr* in *E. coli* From Fattening Pigs and Bovines <12 Months in 2015

In 2015, colistin resistance occurred in 6.5% (14/214) and 3.9% (7/179) of ESBL/AmpC-producing *E. coli* from fattening pigs and bovines <12 months, respectively. Among the indicator *E. coli* population, colistin resistance occurred in 0.6% (1/168) and 4.7% (8/170) of isolates from fattening pigs and bovines <12 months, respectively (**Table 1**). All colistin-resistant *E. coli* from pigs tested positive for *mcr* genes, 14 out of 15 harbored *mcr-*1 and one ESBL-producing isolate tested positive for *mcr-*4 (**Table 1**). Only five out of eight colistin-resistant indicator *E. coli* from bovines yielded *mcr-*1 and one out of eight presented *mcr-*4 (**Table 1**). All seven ESBL/AmpC-producing *E. coli* from bovines yielded *mcr* genes (**Table 1**). Thus, *mcr-*1 was detected in three isolates, *mcr-*3 in three isolates and both *mcr-*1 and *mcr-*3 in one isolate (Supplementary Table 1).

### Genomic Characterisation

High diversity of Sequences Types (STs) was evident when studying the genome of the 28 *E. coli* and three *Salmonella enterica* isolates from turkeys, five *E. coli* from pigs and six *E. coli* from bovine <12 months selected for WGS. In *E. col*i from turkeys, 20 STs were identified. The most represented STs were ST-155 and ST-156 with four isolates each, followed by ST-744 with three isolates and ST-101 with two isolates (Supplementary Table 1). The five *E. coli* from pigs and six *E. coli* from cattle isolates presented different ST. The serotype and the STs of the three *S. enterica* were also different: *S.* Typhimurium ST-3515, *S.* Blockley ST-52 and *S.* Newport ST-45 (Supplementary Table 1).

*mcr-*1.1 (NG_050417.1) was present in 24 isolates: 18 *E. coli* and two *Salmonella* isolates from turkeys, three *E. coli* from pigs and one from bovines. *mcr*-1.2 (KX236309.1) was detected in eight *E. coli* and one *Salmonella* from turkeys. In addition, one *E. coli* isolate from turkey presented a silent mutation, C801T, and two *E. coli* isolates, one from turkey and one from fattening pigs showed a new variant (hereafter termed *mcr-*1.13) of *mcr*-1.1 with two non-synonymous mutations: M2V and S14G. Four *E. coli* from bovines <12 months presented a *mcr-*3 variant, identified as *mcr-*3.2 (NG_055523.1), and one of them presented both *mcr*-1.1 and *mcr*-3.2 (Supplementary Table 1). Also, one ESBL(CTX-M-1)-producing *E. coli* isolate from pigs and one indicator *E. coli* from bovines presented *mcr-*4.2 and *mcr-*4.3, respectively.

The two *E. coli* isolates harboring *mcr-*1.13 allele, ID:14077295 from turkey and ID:15056414 from fattening pig, were genetically different, as shown by MLST (ST-69; ST-5995) and different resistance gene content (Supplementary Table 1). The virulence gene *gad* was detected in both isolates, but it was the only virulence-associated gene found in the pig isolate (Supplementary Table 1). Both isolates harbored the IncF replicon plasmid, but with different plasmid MLST: IncF [F1:A1:B20] and IncF [F46:A-:B42], for the turkey and the pig isolate, respectively (Supplementary Table 1).

All the nine isolates presenting *mcr-*1.2 (IDs:14087995, 14069546, 14062120, 14044802, 14091902, 14045775, 14047606, 14083136, and 14085183) were isolated from turkey. These isolates were one *S.* Blockley, and eight *E. coli* isolates with different STs (Supplementary Table 1). The resistance and virulence genes were variable among those isolates (Supplementary Table 1). For example, one of them (ID:14044802) was only resistant to colistin and only presented the accessory gene *mcr-*1.2, while all other isolates were MDR and ESC-R, and presented ESBL genes such as *bla*_CTX-M-1_ or *bla*_TEM-52_ or other different beta-lactamase genes (Supplementary Table 1). A variety of plasmid incompatibility groups was observed including the plasmids IncFII, IncFI, IncHI2, IncI, IncN in different proportions, but all the nine isolates presented the plasmid IncX4 (Supplementary Table 1). In the isolate ID:14065450, the contig containing *mcr-*1.2 also contained the replicon plasmid IncX4 (contig 35).

The four WGS sequenced isolates harboring *mcr-*3.2 (IDs:15054212, 15038100, 15056874, and 15078696), all of them *E. coli* from bovines <12 months, had different STs and serotype (Supplementary Table 1). All four were MDR and presented the ESBL gene *bla_CTX_*_-M-55_, although always found in contigs other than those containing *mcr-*3.2. The four isolates also shared other resistance accessory genes as *sul3*, *aac*(3)*-Iid*, *aadA2*, or *floR*. A number of different plasmids were detected in all four isolates, with only IncF (F46:A-:B20), IncX1 and IncR detected in all of them. The contigs harboring *mcr-*3.2 did not contain any plasmid replicon type (Supplementary Table 1).

One of the *mcr*-3.2-positive *E. coli* (ID:15054212), presented also *mc*r-1.1. This isolate was ST-744 and contained the following plasmids: IncFIB, IncX4, IncFIC(FII), IncX1, Col156, IncR. *mcr-*1.1 was located in a 32,823 bp contig that included also the replicon sequence of IncX4 (Supplementary Table 1).

The *mcr-*4.2 variant was isolated from an *E. coli* from fattening pig (ID: 15057173-5). This isolate, ST-410, presented the following accessory resistance genes: *bla_CTX-M-1,_ tet(B)-like, sul1,sul2, aadA1, aadB, aph(3^′^)-Ic-*like*, strA, strB, mph(A), floR-*like and *qnrB42-*like (Supplementary Table 1). *mcr-*4.2 and ColE10 replicon sequence were located in the same contig (contig 105; length 7699 bp). In addition to the ColE10 plasmid replicon, this isolate presented other plasmids (Supplementary Table 1).

The *mcr-*4.3 variant was harbored in a *E. coli* (ST-399) isolated from a bovine <12 months (ID: 15050011-1) that also presented other accessory resistance genes as *bla_TEM-1B,_ tet(B), sul1-*like, *sul3, dfrA1, aac(3)-IIa, aadA1, aph(3^′^)-Ic-like, strA, strB* and *catA1-like* (Supplementary Table 1). A variety of plasmids was found, including IncFIA, IncHI1A, IncHI1B(R27), ColE10, IncQ1, ColRNAI, p0111, and Col(MG828), but none in the same contig containing *mcr-*4.3.

### Description of the Contig Harboring *mcr-*1.13

**FIGURE 1:**
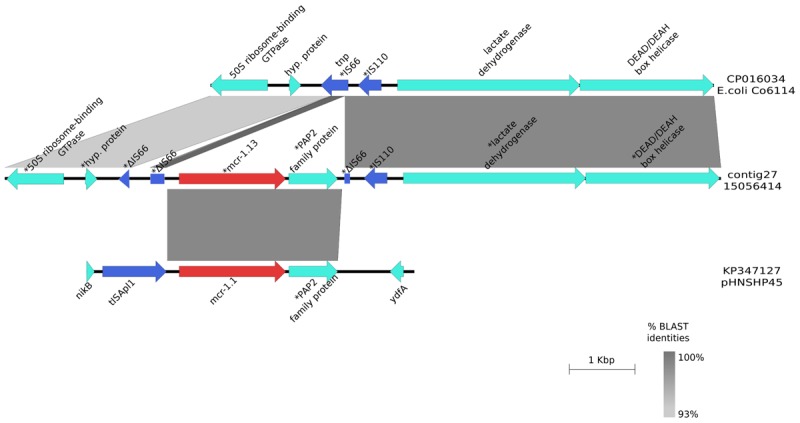
Graphical representation of the *mcr-*1.13 contig. Graphical representation of the BLAST analysis between the contig harboring *mcr-*1.13 from the pig-origin *E. coli* isolate (contig27; 15056414) and the *mcr* plasmid pHNSHP45 (KP347127; region 21000bp- 26000bp) and genomic DNA of *E. coli* Co6114 (CP016034; region 91844bp – 99527bp). ^∗^Indicated that the gene was manually annotated. The gray area represents the blast identities, the percentage of identity is indicated in the legend. Gene colors: red, *mcr*-1; blue, transposases or IS elements.

*mcr-*1.13 was identified in *E. coli* strain 14077295 (contig 564; 10,310 bp lenght) from turkeys and in *E. coli* strain 15056414 (contig 27; 50,840 bp lenght) from pigs. The BLAST alignment of the two contigs showed 99% identity across the entire length of the shortest contig (10,310 bp). No plasmid replicon was detected in any of the two contigs. *mcr-*1.13 (from nt 33,836 to nt 35,462 in contig 27,) was upstream to a PAP2 superfamily hypothetical protein (from nt 35,509 to nt 36,256). Insertion sequences (IS) were detected flanking the *mcr* cassette: upstream *mcr*-1.13 a truncated IS*66* (from nt 33,335 to 33,413 bp and from 33,455 to 33,651 bp) and downstream a small fragment of IS*66* (from nt 36,261 to nt 36,392) and IS*110* (from nt 36,449 to nt 36,816). Upstream the cassette, there was the gene coding for 50S ribosome-binding GTPase family protein (from nt 15,712 to nt 16,627) and downsteam the cassette, there was the coding gene of the subunit YeeA of the methylase (from nt 37,032 to nt 39,849) (**Figure 1**). In BLAST results, contig sequences showed 93–99% identity to *E. coli* chromosome sequence CP016034 except for the region flanked by ISs and containing *mcr-*1.13 and PAP2 superfamily coding genes that had 99 and 100% identity with the same region of the *mcr* plasmid pHNSHP45(KP347127), for the *mcr* gene and the PAP2 superfamily coding gene, respectively (**Figure 1**).

### Description of the Contig Harboring *mcr-*3.2

**FIGURE 2:**
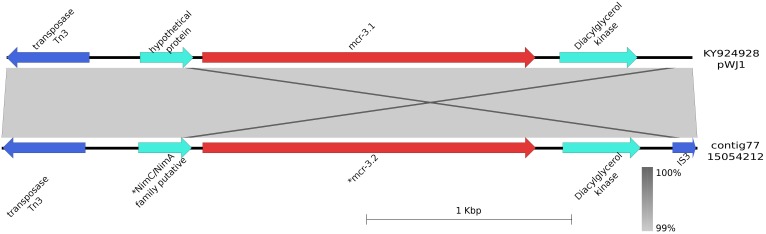
Graphical representation of the *mcr*-3.2 contig. Graphical representation of the BLAST analysis between the contig harboring *mcr*-3.2 in the bovine-origin *E. coli* (contig77; 15054212) and the *mcr* plasmid pWJ1 (KY924928; region 160180 to 163525 bp). ^∗^Indicated that the gene was manually annotated. The gray area represents the blast identities, the percentage of identity is indicated in the legend. Gene colors: red, *mcr*-1; blue, transposases or IS elements.

*mcr-*3.2 was detected in *E. coli* isolates 15054212 (contig 77), 15038100 (contig 128), 15056874 (contig 52), and 15078696 (contig 197) measuring 3,346, 9,285, 5,921, and 5.279 bp, respectively. All contigs were 100% identical to the shortest one. Upstream and downstream *mcr*-3.2 (from 766 to 2,392 bp in contig 77) a diacylglycerol kinase gene (from 268 to 249 bp) and a gene coding for a NimC/NimA putative family protein (from 2,436 to 2,696 bp) were detected, respectively. These three genes were flanked by IS*3* (from 1 to 127 bp) and *Tn*3 (from 2,943 to 3,346 bp) (**Figure 2**). The shortest contig harboring these genes (contig number 77) showed a 99% identity with the pWJ1 plasmid containing *mcr*-3.1 (KY924928) region from 160,180 to 163,525 bp. The genes surrounding the cassette in our strains were not found in the pWJ1 plasmid (KY924928).

### Plasmid Transferability

*E. coli* harboring *mcr-*1.1 (IDs:14043377 and 14065450), *mcr-*1.2 (ID:14047606) and *mcr-*1.13 (ID:14077295), *S*. Typhimurium harboring *mcr-*1.1 (ID:14043372), *S*. Newport harboring *mcr-*1.1 (ID:14038647) and *S.* Blockley harboring *mcr-*1.2 (ID:14085183) were used as donors for conjugation experiments (**Table 2** and Supplementary Table 1). All donors, except the *mcr-*1.13-harboring *E. coli,* transferred *mcr* by conjugation to the *E. coli* K-12 recipient strain. *mcr-*1.1 and *mcr-*1.2 were carried by IncX4 plasmids that transferred either alone or in combination with additional plasmids (**Table 2** and Supplementary Table 1). One isolate (ID: 14065450) displayed in the same contig both *mcr-*1.1 and the replicon of the IncX4 plasmid (contig 68).

**Table 2:** Genotypic traits of donors and transconjugants.

Strain ID	Species	Donors		Transconjugants (*E. coli* K12)	
		AMR genes^1^	Plasmid content^1^	AMR genes^1^	Plasmid content^1^
14043377	*E. coli*	*mcr-*1.1*, bla_TEM-52C_, tet*(A), *aadA1, dfrA1*	IncFII, IncI1, IncFIB, p0111, IncX4	*mcr-*1.1	IncX4
14065450	*E. coli*	*mcr-*1.1*, bla_SHV -12_, sul2*, *sul3*, *aadA1,* *aadA2*, *strA*, *strB*, *aph(3’)-Ia,* *cmlA1*	IncFIB, IncFII, IncI1, IncFIC, IncY, IncQ1, IncX4, Col(MG828)	*mcr-*1.1	IncX4
14047606	*E. coli*	*mcr-*1.2*, bla_CTX-M-1_, tet*(B), *catA1*	IncN, IncX4, Col(MG828)	*mcr-*1.2*, bla*_CTX-M-1_	IncN, IncX4
14077295	*E. coli*	*mcr-*1.13*, bla_TEM-1B_, bla_CTX-M-14_, tet*(B), *sul2*, *strA, strB*, *dfrA14*, *mph*(A)	IncFII, IncFIA, IncFIB, Col156	-	-
14043372	*Salmonella* Typhimurium	*mcr-*1.1*, bla_TEM-1B_, tet*(A), *aadA1*, *aac(3)-IId*	IncI1, IncX4, ColRNAI, Col156, ColpV	*mcr-*1.1*, bla_TEM-1B_, tet*(A), *aadA1*, *aac(3)-IId*	IncI1, ColRNAI, Col156, ColpVC, IncX4
14085183	*Salmonella* Blockley	*mcr-*1.2*, aph(3’)-Ic*, *strA*, *strB*, *mph*(A)	IncN, IncX4, Col156, ColRNAI	*mcr-*1.2	IncX4
14038647	*Salmonella* Newport	*mcr-*1.1*, bla_TEM-1B_, tet*(A), *sul2*, *strA*-like, *strB*, *dfrA14*	IncN, IncX4,ColpVC	*mcr-*1.1	IncX4


*^*1*^Antimicrobial resistance (AMR) genes and plasmid replicons were detected using ResFinder and PlasmidFinder at http://www.genomicepidemiology.org/.*

## Discussion

The high diversity of transferable colistin resistance mediated by *mcr* genes and alleles, quickly spreading across pathogenic enterobacteria globally (Kluytmans, 2017), is an emerging challenge for treatment of Gram-negative infections due to increased occurrence of Healthcare-Associated Extremely Drug Resistant bacterial pathogens (EARS-Net, 2015).

In the present study, we found high prevalence (∼25%) of colistin resistance in both indicator commensal *E. coli* and ESBL/AmpC-producing *E. coli* in turkeys in Italy. In other Italian primary productions such as broilers, fattening pigs and bovines <12 months, colistin-resistant *E. coli* occurred at relatively lower, though still relevant levels.

From a (Veterinary) Public Health perspective, the spread of colistin resistance across production types is of major concern also considering that it was often associated with resistance to multiple drugs and notably also to fluoroquinolones. For instance, MDR *E. coli* exhibiting co-resistance to three Critically Important Antimicrobials for humans were detected in approximately 23% of turkey flocks investigated.

By using WGS, the vast diversity of *mcr* types and variants was evident within and across the major production types. At least three *mcr-*1 alleles were identified in turkeys, pigs and bovines <12 months in a 2-year-period, one of which has not been previously described. The majority of isolates harbored *mcr*-1.1, but *mcr-*1.2, *mcr-*1.13, *mcr-*3.2, *mcr*-4.2 and *mcr*-4.3 were also detected.

The core genomes of the *E. coli* and *Salmonella* isolates harboring *mcr-*1 were quite variable as shown by diversity of MLST types, in agreement with what has been observed in different contexts previously (Hasman et al., 2015; Bernasconi et al., 2016; Cannatelli et al., 2016; Zhao et al., 2017).

Conjugation experiments and WGS data analysis confirmed that *mcr-*1.1 and *mcr-*1.2 were located on IncX4 plasmids in *E. coli* and *Salmonella* spp. from turkey. In Europe, IncX4 plasmids have been described in association with *mcr-*1 in isolates of animal origin in Belgium (Xavier et al., 2016; Garcia-Graells et al., 2017) and from humans in Italy (Di Pilato et al., 2016).

The *mcr-*1.2 gene was first described on an IncX4 plasmid in a *Klebsiella pneumoniae* isolated from a human sample in the Tuscany Region (Italy) (Di Pilato et al., 2016). In our study, this variant was found on IncX4 plasmids in intestinal *Salmonella* and *E. coli* spread throughout the Italian fattening turkey production. Our observations suggest the transmission of *mcr*-positive IncX4 plasmids between different bacterial species, with the possibility of transmission from animals to humans, or vice versa.

The *mcr-*3 gene was first described on an IncHI plasmid (Yin et al., 2017), whereas the *mcr-*3.2 variant has been described from the whole genome shotgun sequence of a *Shigella sonnei* strain (NG_055523; NPZH01000177) isolated in the United States, but to our understanding, no formal description has been published to date. In our study, four ESBL/AmpC-producing *E. coli* isolates from bovines <12 months presented *mcr-*3.2. The WGS data analysis performed on these isolates did not allow us to determine the genomic location of *mcr-*3.2. However, only one of these isolates harbored an IncHI plasmid. A ST heterogeneity of *E. coli* isolates harboring the *mcr-*3.2 gene was evident. Further conjugation or transformation experiments are needed in order to elucidate if the *mcr-*3.2 found in *E. coli* in Italian cattle is plasmid-borne and transferable.

One *E. coli* from cattle was simultaneously positive for *mcr*-3.2 and *mcr*-1.1. In this isolate, *mcr-*1.1, was located on an IncX4 plasmid. An *E. coli* carrying both *mcr*-1.1 and *mcr*-3.2 and also isolated from cattle has been recently described in Spain (Hernández et al., 2017). Interestingly, all four *mcr-*3.2-positive *E. coli* isolated detected in bovines <12 months in Italy carried the ESBL *bla*_CTX-M-55_ gene, apparently an emerging variant in veal calves in Italy. The same observation has been made by other authors (Hernández et al., 2017; Roer et al., 2017). Taken together, these features could be suggestive of a genetic linkage between the two genes. Our study, however, provides preliminary data that the *mcr*-3.2 and the *bla*_CTX-M-55_ genes may not be linked on the same genetic element, and that this aspect needs further investigation.

The *mcr-*4.2 gene was described recently from two *S. enterica* Typhimurium (monophasic variant) isolated from human samples in Italy (Carretto et al., 2018). So far, this variant has not been associated to any plasmid. In our study, the *mcr-*4.2 gene identified in a ESBL(CTX-M-1)-producing *E. coli* isolated from a fattening pig, was located on a ColE plasmid, exactly the same type of plasmid in which *mcr-*4.1 was described by Carattoli et al., 2017. The *mcr-*4.3 variant was first described from a *Salmonella* Kedougou isolated from pigs in Spain (Rebelo et al., 2018). In our study, *mcr-*4.3 was harbored by an indicator *E. coli* from a bovine caecal sample. All the *mcr-*4 variants described so far have been found in *Salmonella* or *E. coli* from animals and from humans, despite their localisation on non-conjugative plasmids, similarly to what has been found with *mcr-*4.1 by Carattoli et al. (2017).

The *mcr*-1.13 gene was located on a mobile genetic element inserted in the chromosome. The *mcr* cassette found is different from the chromosomal *mcr* cassettes described so far, because of the presence of ISs other than IS*plA* (Snesrud et al., 2016; Li et al., 2017). By considering the high rate of self-transferability of the IncX plasmids (Sun et al., 2017) and that IncX4 is dominant in the Italian primary productions surveyed, this finding suggests that the *mcr* cassette may have been acquired from an IncX4 plasmid which lacks IS*plA* insertion sequence (Bernasconi et al., 2016; Matamoros et al., 2017; Sun et al., 2017). It is likely that the insertion occurred in different moments, since the two *E. coli* isolates derived from two different animal productions and presented different genome content. The ability of the *mcr* cassette to jump into several types of plasmids (IncI2, IncX4, IncHI2, and IncP) or into the chromosome is highly concerning also in view of the possibility of insertion into plasmids already present in MDR isolates, or even of the creation of a new mega-plasmid as occurred in *Salmonella* Infantis (Franco et al., 2015). This would imply that colistin resistance could be co-selected by the use of others antimicrobials. Indeed, multidrug resistance in colistin-resistant, *mcr*-positive isolates is a constant feature we have observed in these population-based studies. These findings highlight how rapidly mobile genetic elements can be acquired by *Enterobacteriaceae* and how genes can mutate in environments with high selective pressure as that occurring in the intensive farming systems. In this respect, the selection pressure exerted by the use of colistin and the complex co-selection mechanisms triggered by the overall high use of several antibiotic classes in the meat-producing industry is likely to have played a major role. For instance, the overall exposure to antibiotics in food-producing animals in Italy in 2014 was estimated around 360 mg/Population Correction Unit (PCU, i.e., mg per kilogram of biomass of farmed animals), and the exposure to colistin only was estimated around 29 mg/PCU (ESVAC, 2016). The mean, median and range of total sales in Europe (29 countries) were 108, 66, and 3 – 419 mg/PCU, respectively, while 22 out of 29 countries reported sales of colistin ≤5 mg/PCU (range of the 29 EU countries: 0.06–36.10 mg/PCU). It is well known that the intensively farmed, meat-producing animals (turkeys, broilers, pigs, bovines <12 months) are the population categories at risk of exposure to higher amounts of colistin in modern farming systems, and the results of this study strongly support the general concept that the spread of *mcr*-mediated colistin resistance has been favored by the semi-continuous and high exposure to colistin in these production chains.

Whether animals are an important source for human extraintestinal pathogenic *E. coli* (ExPEC) infections in humans is still a matter of debate (Bélanger et al., 2011). Few studies provide information and comparison on STs and plasmids harboring ESBLs or transferable AmpCs (Leverstein-van Hall et al., 2011) for both human and food-producing animal isolates. Although conclusive epidemiological evidence is still lacking, it has been proposed that some human ExPEC infections could arise from poultry and pig ExPEC reservoirs (Jakobsen et al., 2010). That being said, it is interesting to notice that at least one-fourth of *E. coli* isolates described in our study belong to the same STs as isolates associated with human ExPEC infections in Europe. In some cases (Leverstein-van Hall et al., 2011; Mavroidi et al., 2012; Brolund et al., 2014) they also share the same ESBL (e.g., ST69 and CTX-M-14, ST410 and CTX-M-1). As for colistin-resistant *E. coli*, one of the *mcr*-1.1 positive isolates from turkeys here described belongs to ST131, although it lacks the virulence gene markers and ESBL generally associated with the globally spread human clinical clone. Indeed, a *mcr*-1-positive, ESBL-negative ST-131 *E. coli* was also described in human bloodstream infections in Italy in 2017 (Corbella et al., 2017). Overall, these observations should be taken very cautiously, since methods for genome analysis and parameters for assessing relatedness among both core genomes and accessory genomes are quickly evolving, which implies that it may be necessary to re-evaluate any earlier conclusions on relatedness or source attribution based on partial molecular characteristics, as previously shown (De Been et al., 2014).

## Conclusion

In conclusion, harmonized cross sectional studies at slaughter like the ones implemented by the EU represent a very important tool for a deep insight into trends and emergence of antimicrobial resistance traits and patterns in major food-borne pathogens and commensal opportunistic bacteria. Especially when occurring at high prevalence, the spread of transferable colistin resistance in *E. coli* (both indicator commensal and ESBL/AmpC-producing isolates) is to be considered a concern *per se*. Additionally, as a general principle, the high spread of resistance increases the probability of transfer of specific resistance traits also to major zoonotic pathogens, such as *Salmonella* spp. The hypothesis that horizontal transfer, so far, has played a major role in spread of colistin resistance among bacteria in Italian meat-producing animals is supported by the observed heterogeneity of *mcr-*positive *E. coli.* Indeed, at least in the Italian turkey productions, we demonstrated that the same transferable determinant of colistin-resistance is being carried on the same conjugative plasmid in both *E. coli* and major *Salmonella* serotypes detected in the same intensive-farming industry.

For the above reasons, quick and thorough action should be taken by the farming industry and by the Competent Authorities to drastically reduce the use of colistin in food-producing animals, especially in turkeys, following the recommendations of the European Medicines Agency (≤5 mg/PCU). EU Member States were encouraged to set stricter national targets, ideally below 1 mg/PCU colistin. We also strongly recommend reducing the overall use of all other classes of antibiotics at primary production level, in order to mitigate the effects of the complex mechanisms behind co-selection and multidrug resistance toward Critically Important Antimicrobials, in a “Consumer Protection” and a “One Health” perspective.

## Author Contributions

PA, PL, AF, RH, VB, and AB conceived and designed the experiments. PA, PL, FF, AI, and FS performed the experiments. PA, AF, FF, AI, RH, VB, and AB analyzed the data. PA, PL, AF, AC, RH, VB, and AB contributed reagents, materials, and analysis tools. PA, AF, RH, VB, and AB wrote the paper.

## Conflict of Interest Statement

The authors declare that the research was conducted in the absence of any commercial or financial relationships that could be construed as a potential conflict of interest.

## Disclaimer:

The conclusions, findings and opinions expressed in this scientific paper reflect only the view of the authors and not the official position of the European Food Safety Authority.

## Footnotes

1 http://eur-lex.europa.eu/legal-content/EN/TXT/HTML/?uri=CELEX:32013D0652&from=IT

2 https://www.eurl-ar.eu/CustomerData/Files/Folders/21-protocols/276_esbl-ampc-cpeprotocol-version-caecal-january2017-version4.pdf

3 http://www.ebi.ac.uk/ena

4 http://www.genomicepidemiology.org/

5 https://blast.ncbi.nlm.nih.gov/Blast.cgi
